# Impact of CD133 positive stem cell proportion on survival in patients with glioblastoma multiforme

**DOI:** 10.2478/raon-2013-0055

**Published:** 2013-10-08

**Authors:** Marju Kase, Ave Minajeva, Kristi Niinepuu, Sandra Kase, Markus Vardja, Toomas Asser, Jana Jaal

**Affiliations:** 1 Faculty of Medicine, University of Tartu, Estonia; 2 Riga Stradinš University, Riga, Latvia; 3 Hematology and Oncology Clinic, Dept. of Radiotherapy and Oncological Therapy, Tartu University Hospital, Estonia; 4 Neurology Clinic, Dept. of Neurosurgery, Tartu University Hospital, Estonia

**Keywords:** glioblastoma multiforme, CD133, stem cells, radiotherapy, survival

## Abstract

**Background:**

The aim of the study was to assess the impact of CD133-positive (CD133+) cancer stem cell proportions on treatment results of glioblastoma multiforme (GBM) patients.

**Patients and methods:**

Patients with GBM (n = 42) received postoperative radiotherapy (± chemotherapy). Surgically excised GBM tissue sections were immunohistochemically examined for CD133 expression. The proportions of CD133+ GBM cells were determined (%). The proportion of CD133+ GBM stem cells was established by 2 independent researchers whose results were in good accordance (R = 0.8, p < 0.01). Additionally, CD133 expression levels were correlated with patients overall survival.

**Results:**

The proportion of CD133+ cells varied between patients, being from 0.5% to 82%. Mean and median proportions of CD133+ cells of the entire study group were 33% ± 24% (mean ± SD) and 28%, respectively. Clinical data do not support the association between higher proportion of stem cells and the aggressiveness of GBM. Median survival time of the study group was 10.0 months (95% CI 9.0–11.0). The survival time clearly depended on the proportion of CD133+ cells (log rank test, p = 0.02). Median survival times for patients with low (< median) and high (≥ median) proportion of CD133+ cells were 9.0 months (95% CI 7.6–10.5) and 12.0 months (95% CI 9.3–14.7), respectively. In multivariate analysis, the proportion of CD133+ cells emerged as a significant independent predictor for longer overall survival (HR 2.0, 95% CI 1.0–3.8, p = 0.04).

**Conclusions:**

In patients with higher stem cell proportion, significantly longer survival times after postoperative radiotherapy were achieved. Underlying reasons and possible higher sensitivity of GBM stem cells to fractionated radio-therapy should be clarified in further studies.

## Introduction

Central nervous system (CNS) cancers are generally considered rare tumours. However, according to recent epidemiological study, there are about 27700 new CNS tumour cases each year in Europe.^1^ Glioblastoma multiforme (GBM) is the most aggressive and rapidly fatal type of a brain tumour in adults that accounts for approximately 20% of all malignant primary CNS cancers.^2^ In spite of decades of intensive research, the prognosis of patients with GBM is still poor with a median survival time up to 14.6 months.^3^ Since 1978, local radiotherapy, administered after debulking surgery, has been a mainstay of standard treatment of GBM patients.^4^ Although radiotherapy results in excellent local control and cure rates in most solid tumours, the efficacy of this treatment modality in GBM is limited. Almost all GBM patients develop fast disease progression and tumour recurrence within or immediately adjacent to the high-dose radiation volumes.^5^ Therefore, GBM is by nature one of the most radioresistant tumours that represents a big challenge in neuro-oncology.

Detailed information about molecular mechanisms of radioresistance of GBM is not known. However, previous studies have shown that radioresistance may involve many tumour cell and surrounding microenvironment processes, including changes in growth factors, receptors, different signalling and apoptotic pathways and DNA repair mechanisms.^6^,^7^ Additionally, previous *in vitro* and *in vivo* studies have proposed that CD133 positive (CD133+) tumour cells represent the cellular population that confers GBM radioresistance and could therefore be the source of tumour recurrence after radiation.^8^ CD133 is a transmembrane glycoprotein which is expressed in different type of progenitor cells, including hematopoietic stem cells. In GBM, CD133+ cells are considered stem cells because of their ability to self-renew, differentiate and to initiate tumour formation *in vivo*.^9^

Whether higher proportion of CD133+ GBM cells (stem cell population) contributes to worse treatment results after postoperative radiotherapy was tested in the present study.

## Material and methods

Between January 2006 and December 2008, 42 patients with GBM were treated with postoperative three-dimensional radiotherapy at Tartu University Hospital or North Estonian Medical Centre. Characteristics of patients are listed in Table 1.

### Radiotherapy treatment planning and treatment parameters

Treatment planning was performed using CT/MRI scans and TPS XiO CMS treatment planning system. The gross tumour volume (GTV) encompassed the resection cavity and any residual tumour. A 2–3 cm margin was added to create clinical target volume (CTV). Critical tissues were spared (brainstem, chiasma). For planned target volume (PTV), 0.5 cm margin was included. Treatments were performed using linear accelerators (30–60 Gy in 2.0 Gy fractions; mean dose 54 Gy). The prescribed dose was normalized to 100% at the isocenter and PTV was covered by 95% isodose surface (ICRU Report 50). None of the patients received concomitant and adjuvant chemotherapy with temozolomide (available in Estonia since 2010). However, for recurrent disease, 26 patients received chemotherapy with lomustine (CCNU).

### Immunohistochemistry (IHC)

Haematoxylin and eosin stained sections (4 μm thick) were used for primary diagnosis. The diagnosis of GBM was confirmed by 2 independent pathologists.

Additional sections were cut from archived paraffin blocks and stained according to standard IHC protocol. For immunohistochemistry, a primary antibody against CD133 was applied (Biorbyt Ltd., #orb18124, United Kingdom, dilution 1:50). Diaminobenzidine was used as chromogen.

The evaluation and scoring of slides were carried out in a blinded fashion by 2 independent researchers. The proportion of CD133+ cells was determined in randomly taken microscopic fields at a magnification of 400. For individual values, the mean of 6 microscopic fields was calculated (%). The proportion of CD133+ GBM cells was determined in areas with vital tumour tissue. Additionally to CD133+, the overall proportion of necrosis (%) was determined in haematoxylin-eosin stained tissue sections by an experienced pathologist.

Individual means of CD133+ cell proportions were used to determine group median value. According to the median value of CD133+ proportions, patients were divided into subgroups < median (less than median) and ≥ median (equal and more than median). These groups were used in survival analysis.

## Statistical analysis

The SPSS statistical software was used to calculate individual means, group mean, and standard deviation of the mean, as well as median value. Additionally, Pearson correlation analysis was utilized. The proportion of pre-irradiation CD133+ GBM cells was correlated with the overall survival (OS) that was defined as the period from the date of operation to the date of death resulting from GBM or to the date of last analysis. Survival curves were created using the Kaplan-Meier method and differences between the groups were compared using the log-rank test. Multivariate analysis was performed using the Cox proportional hazards model. A p-value < 0.05 was regarded statistically significant.

Study was carried out with permission of Research Ethics Committee of the University of Tartu. The study was carried out according to the Declaration of Helsinki.

## Results

### Proportion of CD133+ GBM cells

**FIGURE 1.**
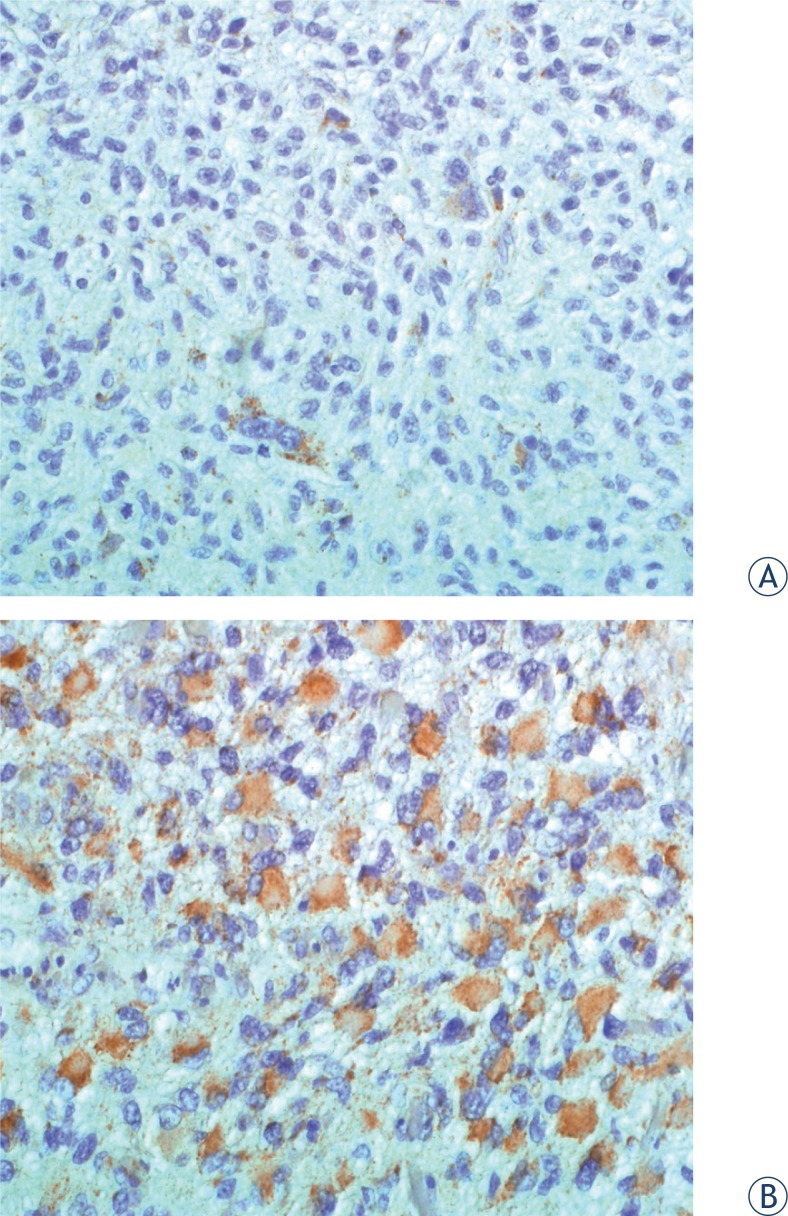
Different proportions of CD133+ stem cells (1A: low, 1B: high) in glioblastoma multiforme.

In GBM tumour samples, the proportion of CD133+ cells varied greatly between patients. Figure 1 illustrates low (< median) and high (≥ median) CD133+ cell proportions in tumour tissue. The proportion of CD133+ GBM stem cells was determined by 2 independent researchers whose results were in good accordance (R = 0.8, p < 0.0001). Among individual GBM patients (n = 42), the proportion of CD133+ stem cells in tumour tissue ranged from 0.5% to 82% (individual means). Mean and median proportions of CD133+ cells of the entire study group were 33% ± 24% (mean ± standard deviation) and 28%, respectively. According to individual values, patients were divided into two groups: patients with low (< median) and high (≥ median) proportion of CD133+ GBM cells. Groups were sufficiently balanced, since there were 20 patients (48%) with low (< median) and 22 patients (52 %) with high (≥ median) proportion of CD133+ cells.

Additionally to immunohistochemistry, the overall proportion of necrosis (%) was determined in haematoxylin-eosin stained tissue sections. The mean proportion of necrosis of the entire study group was 38 ± 31% (mean ± standard deviation). Correlation analysis, based on individual values, revealed a significant association between the proportion of stem-cells and the percentage of necrosis (p < 0.01).

### Correlation of CD133+GBM cell proportion with overall survival

**FIGURE 2.**
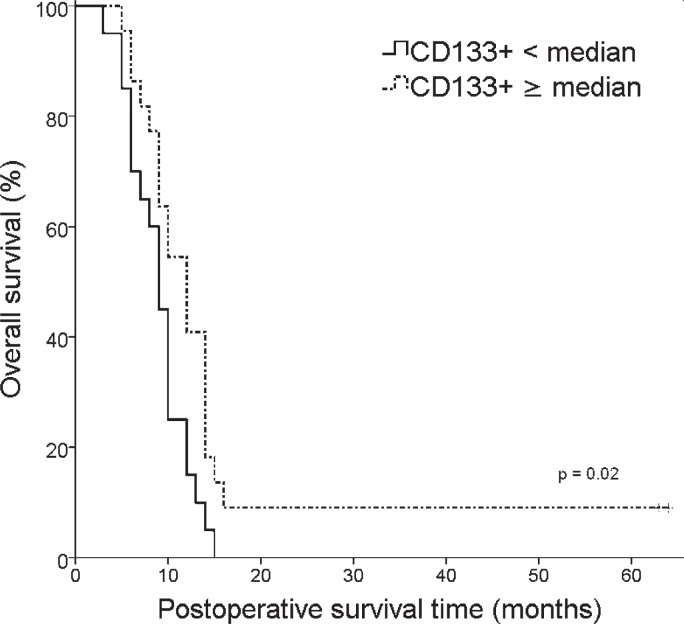
Kaplan-Meier analysis of overall survival (OS) according to CD133+ GBM stem cell proportions (< median *vs* ≥ median).

At the time of analysis 40 patients had died. The median OS of the whole study group was 10.0 months (95% CI 9.0–11.0). Figure 2 illustrates the OS among patients with low and high proportion of CD133+ GBM cells. The survival times clearly depended on the proportion of CD133+ cells (log rank test, p = 0.02). Median survival times for patients with low (< median) and high (≥ median) proportion of CD133+ cells were 9.0 months (95% CI 7.6–10.5) and 12.0 months (95% CI 9.3–14.7), respectively.

In multivariate analysis (Table 2), the proportion of CD133+ cells (HR 2.0, 95% CI 1.0–3.8, p = 0.04) and Karnofsky performance score (HR 2.2, 95% CI 1.0–4.8, p = 0.04) emerged as significant independent prognostic factors for OS.

## Discussion

The molecular basis of radioresistance of GBM is not known. Therefore, a precise knowledge about underlying mechanisms is essential to develop clinically useful methods to radiosensitize and treat this incurable disease. Previous *in vitro* and *in vivo* studies have proposed that CD133+ tumour cells represent the cellular population that confers GBM radioresistance and could therefore be the source of tumour recurrence after radiation.^8^

According to the brain tumour cancer stem cell model, a subpopulation of cancer cells possesses the capacity of self-renewal, tumour formation and the capability to form progeny with a more restricted fate.^10^ In GBM, several stem cell candidate markers have been explored, however, out of these, CD133 is the most studied.^7^,^11^ CD133+ GBM cells are considered stem cells because of their ability to self-renew, differentiate and to initiate tumour formation *in vivo*.^9^ An injection of as few as 100 CD133+ cells has been shown to produce a tumour that could be serially transplanted and which was phenotypically resembled the patients original tumor.^9^

In the current study, the presence of CD133+ cells in GBM tissue was detected by immunohistochemical staining method. The proportion of CD133+ GBM stem cells was determined in surgically excised tumour tissue, *i.e.* prior radiotherapy. The study revealed wide variability in the proportion of these cells. Among evaluated GBM samples, there were tumours that contained only 0.5% CD133+ GBM cells but also tissues in which the proportion of CD133+ cells was as high as 82%. The variability in CD133+ GBM stem cell proportions has also been reported in studies of 37 and 44 consecutive GBM patients, where CD133 expression ranged between 0.5% and 10.0%^12^,^13^ However, in our study, somewhat higher CD133+ GBM cell proportions were detected (median 28%) that might be related to the use of different primary CD133 antibody clone.^14^

Present study revealed the correlation between the proportion of CD133+ stem cells and the overall proportion of tissue necrosis. It is widely accepted that necrosis typically develops in hypoxic (low-oxygen) environments. In GBM, the expression of hypoxia markers (carbonic anhydrase IX [CAIX] hypoxia inducible factor-1 [HIF-1α]) has been shown to be especially high in tumour regions containing 10% to 45% necrosis of total area.^15^ Additionally, it has been reported that tumour-initiating CD133+ GBM stem cells are preferentially expanded in hypoxic conditions.^15^,^16^ Therefore, hypoxia might have also influenced the proportion of CD133+ GBM cells in the present study.

Additionally to the determination of CD133+ cell proportions, tumour CD133 expression levels were correlated with GBM patients overall survival. The median survival of the entire study group was 10.0 months. This is in a good accordance with previous studies where postoperative radiotherapy has resulted in median survival of 9–11.6 months.^4^,^17^ However, the survival time clearly depended on the proportion of CD133+ GBM stem cells. Median survival times for patients with low (< median) and high (≥ median) proportion of CD133+ cells were 9.0 months and 12.0 months respectively. In contrast to what was expected, significantly longer survival times after postoperative radiotherapy were achieved in patients with higher stem cell proportion. To the knowledge of authors, there are no other clinical studies that would have evaluated the prognostic significance of CD133 expression after GBM radiotherapy. Nevertheless, clinical series that have used radiochemotherapy (radiotherapy and concomitant plus adjuvant temozolomide), which currently represents standard-of-care treatment for GBM, have shown opposite results. In clinical study of 44 GBM patients, the CD133+ tumour cell proportion of ≥ 2% negatively correlated with overall survival.^12^ Additionally, mRNA expression analyses in GBM patients showed that high sample CD133 mRNA expression was a significant prognostic factor for adverse overall survival.^18^,^19^ These opposite results may be related to other treatment protocol (radiochemotherapy), different primary antibody used for CD133 immunohistochemical detection, as well as to the fact that mRNA expression study samples contained up to 50% of non-tumour tissue, which may also have contained CD133.^20^

Similarly to our findings, different clinical outcomes were documented in a study that divided GBM patients into 2 groups (CD133-low, CD133-high) according to CD133+ cell ratio either < 3% or ≥ 3%, as detected by the fluorescence activated cell scanning (FACS) analysis of primary tumour cultures. Namely, tumours from CD133-low GBM patients were shown to have tendency to be localized within the deeper structures of the brain, to show more invasive growth patterns and ventricle involvement, as well as relatively higher rate of disease progression after radiotherapy and chemotherapy.^21^ Also, although not in primary GBM, significantly longer survival times were detected in recurrent GBM patients with higher proportion of CD133+ cells.^13^ In addition, the multivariate analysis of the present study revealed that next to the well-established prognostic factor Karnofsky performance status (KPS), CD133+ GBM stem cell proportion emerged as a significant independent predictor for overall survival. This clearly suggests that GBM patients with high proportion of CD133+ tumour cells respond better to radiotherapy and achieve better treatment response that consequently result in longer survival times.

It has been widely accepted that CD133+ GBM stem cells are especially radioresistant.^8^ The findings of our study point toward the possibility that these cells might be, in contrast to what has been believed, radiosensitive. The radioresistant nature of CD133+ GBM stem cells has been mainly documented in studies that compare isolated CD133+ and CD133− GBM cell lines.^8^,^22^ However, when compared to the traditional glioblastoma established cell lines that contain heterogeneous cell subpopulations, higher radiosensitivity of CD133+ GBM stem cells has been seen. It has been previously reported that CD133+ GBM stem cells have a reduced capacity to repair radiation-induced double strand brakes, which is likely to be a major contributor to the relatively greater degree of radiosensitivity.^23^ Therefore, the radiosensitivity of CD133+ GBM stem cells might be greatly underestimated.

As mentioned earlier, a correlation between the proportion of CD133+ GBM stem cells and the overall proportion of tissue necrosis was found. This shows indirectly that also a surrounding microenvironment may contribute to the radiation response of GBM stem cells. Indeed, recent publications have confirmed this relationship. It has been demonstrated that GBM stem cells irradiated *in vivo* within orthotopic xenografts are less susceptible to double strand brakes induction and have greater capacity to repair damage as compared to same tumour cells irradiated under *in vitro* growth conditions.^24^ Moreover, close correlation between CD133+ GBM cells and hypoxia^15^, vascular structures^25^, extracellular matrix (ECM) components^7^, as well as inflammation and immunoregulatory markers^26^ have been reported. This all shows that radiation response of CD133+ GBM stem cells are determined by a numerous known and unknown processes that can be collectively named as “micro-environment-stem cell unit”.^7^

The present study has several limitations. These include retrospective data collection and small number of patients. Also, some important variables, such as tumour O6-methylguanine-DNA methyltransferase (MGMT) methylation status, isocitrate dehydrogenase 1 (IDH1) gene mutation status, recursive partitioning analysis (RPA) and patient’s quality of life scores were not recorded. However, this small study showed that there is no association between higher proportion of stem cells and the aggressiveness of GBM. In contrast, in patients with higher stem cell proportion, significantly longer survival times after postoperative radiotherapy were achieved. Since radiotherapy is one of the main treatment modalities in GBM, further studies are needed to clarify these results for better understanding of GBM biology that consequently may result in more effective treatment methods to fight this devastating disease.

## Figures and Tables

**TABLE 1.** Characteristics of 42 patients with glioblastoma multiforme

**Variable**	**No of patients (n = 42)**	**Percentage (%)**
Gender		
• Male	23	55%
• Female	19	45%
Age, years (range)^*^	30–77	
Radiotherapy dose (range)	30–60 Gy	
Chemotherapy^**^		
• No	16	38%
• Yes	26	62%

* Age at the time of operation;

** Used for recurrent disease

**TABLE 2.** Multivariate analysis for overall survival (OS)

**Variable**		**OS**	
		**p**	**HR (95% CI)**
CD133+	< median *vs* ≥ median	0.04	1.99 [1.04–3.83]
Radiotherapy dose^*^	range 30–60 Gy	0.24	0.96 [0.90–1.03]
Chemotherapy	yes *vs* no	0.75	1.13 [0.54–2.37]
Karnofsky performance score	< 70% *vs* ≥ 70%	0.04	2.24 [1.04–4.83]

* Continuous variable; HR = hazard ratio; CI = confidence interval
